# Control of Machining of Axisymmetric Low-Rigidity Parts

**DOI:** 10.3390/ma13215053

**Published:** 2020-11-09

**Authors:** Antoni Świć, Arkadiusz Gola, Łukasz Sobaszek, Olga Orynycz

**Affiliations:** 1 Faculty of Mechanical Engineering, Lublin University of Technology, Nadbystrzycka 36, 20-618 Lublin, Poland; a.swic@pollub.pl (A.Ś.); l.sobaszek@pollub.pl (Ł.S.); 2 Department of Production Management, Bialystok University of Technology, Wiejska Street 45A, 15-351 Bialystok, Poland; o.orynycz@pb.edu.pl

**Keywords:** low-rigidity, stiffness, machining, control, cutting force, stability

## Abstract

The specific character of the process of machining of axisymmetric low-rigidity parts makes it difficult to obtain finished products with a required accuracy of shape and dimensions and surface quality. The methods traditionally used to achieve accuracy in the machining of low-rigidity shafts considerably reduce the efficiency of the process, fail to meet modern automation requirements, and are uneconomical and not very productive, which means new methods for controlling the machining of low-rigidity shafts need to be looked for. This article presents a structural and a calculation scheme of a machining system for the turning of low-rigidity parts and a control model based on the second-order Lagrange equation. The first section of this paper presents qualitative relationships among variables in the proposed technological system for machining axisymmetric low-rigidity parts. Moreover, schematic of the machining system for the processing of such parts as well as equations describing the energy state of the machining system is presented. Next, mathematical model of optimal system control during the machining process, which permits to control a system under specific conditions and obtains a higher shape accuracy were introduced. The key stage of the verification process concerns the numerical validation of proposed solutions. Experimental studies confirm that the utilization of the proposed mathematical models describe the properties of the original object with sufficient accuracy and allow to obtain a higher machined shaft shape accuracy.

## 1. Introduction

In the machine industry, axially symmetrical parts make up about 34% of the total production. Twelve percent of those are low-rigidity shafts [1]. Low-rigidity shafts are used in the aerospace industry, precision mechanics, tool-making industry (special tools), and automotive industry [2,3,4]. They are characterized by an irregular shape and low stiffness in specific cross-sections and directions. During production, high requirements are placed on their geometric parameters, mutual position of the surfaces, linear dimensions, and surface quality [5].

Machining accuracy depends, to a large extent, on the compatibility between workpiece stiffness and the stiffness of the machine tool, and can be subject to large variation [6]. In a specific technological system, a low-rigidity workpiece is the system’s weakest element [7,8]. Researchers make attempts to identify important machining process factors and develop effective control methods. Taranenko et al. [9] proposed to consider low-rigidity shafts as control objects with predetermined parameters. The technological system is understood as a sequence of the following components: headstock–low-rigidity shaft–tailstock, and a center or a clamp [10]. Vinayagamoorthy and Xavior [11] indicate the need of multi-objective approach consideration in the machining process and suggest the mathematical description of the control object. Świć et al. in the paper [12] determine and describe the deformation function of a low-rigidity shaft—one should take account of factors such as the work-holding method and the loading conditions.

The specific nature of the process of machining of low-rigidity shafts makes it difficult to obtain finished products with a required accuracy of shape and dimensions and surface quality [13]. Under specific conditions, the low inherent rigidity of the shaft and its relatively low stiffness in comparison with the rigid assemblies of the machine tool may lead to vibrations [14]. Qi et al. in the paper [15] presented a method based on the machining forces prediction to achieve appropriate process control. Another approach to the problem state solution proposed by Pahar et al. [16]—authors consider vibration as a nonlinear natural vibration and describe process by means appropriate equations. Ma et al. [17] proposed a dedicated system that utilized strain measurement to control vibrations and other important shafts machining parameters. Huang et al. except vibration influence, stressed on the need to consider other factors [18].

The need to obtain appropriate surface quality parameters while ensuring a high machining performance spurs the search for new methods of producing long low-rigidity shafts, using machining parameters similar to those used in the production of parts with normal stiffness [13,19]. 

Machining is affected by numerous interfering factors that destabilize the process (large deformations of the workpiece, the machine tool, and the fixture; swarf, dust, etc.,) [20,21], compromising the quality of the parts produced [22,23]. Arnaud et al. in [24] presented simulation-based techniques to analyze and control low-rigidity turning process. Urbicain et al. proposed stability lobes utilization to increase process stability [25]. Gao et al. made attempts to solve mentioned problems by means of automated approach based on point cloud registration and current IT hardware utilization [26]. Lopes et al. [27] developed matrix-based model to take into account the multivariate uncertainty of the turning process. 

Abhishek and Karthik [28] outlined the important aspects of developed method which could be utilized in real manufacturing systems. Measures traditionally used to achieve accuracy in the machining of low-rigidity shafts, which involve multi-pass machining, lowering of the machining parameters, use of steadies, use of additional treatments, and manual lapping, substantially reduce the efficiency of the process, and in many cases fail to produce the expected result [29]. They also do not meet modern automation requirements and are uneconomical and inefficient [30,31]. This means that new methods of controlling the machining of low-rigidity shafts have to be developed.

In this paper, authors made attempts to develop the new method of axisymmetric low-rigidity parts machining control. In response to the emerging in the literature needs the authors describe a machining system for the turning of low-rigidity shafts and formulate a control model based on the second-order Lagrange equation. Developed solution can be implemented in intelligent machining-process control systems.

Section 2 summarizes the essential information regarding control object in the machining of elastically deformable shafts. The new model of a machining system for low-rigidity parts is described in Section 3, and the mathematical modelling, evaluation, and results are discussed in the subsequent section. Conclusions and plans for further research work are presented in the last section of the work. 

## 2. Characteristics of the Control Object in the Machining of Elastically Deformable Axisymmetric Parts

**Figure 1:**
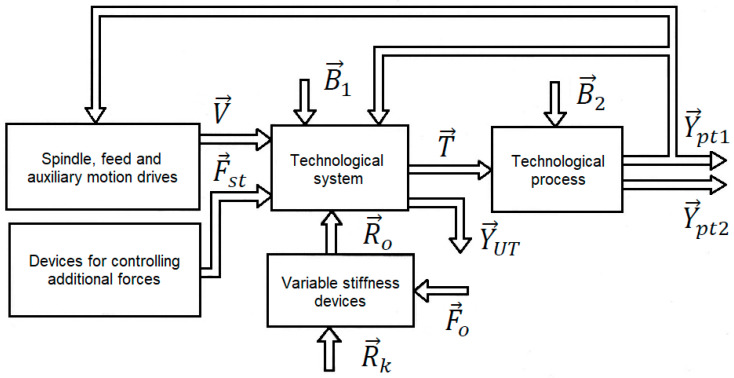
Schematic of a machining system for the processing of low-rigidity parts.

Parts are processed using machine tools in accordance with the technological processes (TP) developed at the stage of technical preparation of production. Active control of a TP allows to increase the accuracy of the shape and dimensions of parts, reduce surface roughness, improve the technical and economic performance of the process, and increase the accuracy of TS. Therefore, when controlling an object of this type, it is necessary to take into account the processes occurring in the machining zone and in the TS terminating with the machining process. In particular, the widest possible range of changes in the parameters of the control object must be taken into account. Because, in a TS, constraints have a variable nature, the parameters may change during the processing of one part. The qualitative relationships among variables in a machining system are shown in Figure 1.

In accordance with the schematic shown in Figure 1, the generalized vector of the possible regulated coordinates of the TS can be denoted as:

(1) $${\vec{Y}}_{UT}=\left({\vec{g}}_{UT},d_{cz},H_{cz},W_{cz},e_{cz},\ldots \right)$$

The most important output variables are the elastic deformations of the TS, which are characterized by vector ${\vec{g}}_{UT}$. The variability of elastic deformations of the TS of the machine tool causes changes in the spatial position of the cutter and the actuating (end) parts of the TS, which leads to a reduction in machining accuracy. Other variables that can be adopted as output variables for machine tool systems are those that directly characterize the accuracy and quality of machining. They include the diameter of the part $d_{cz}$, its surface roughness $H_{cz}$ and waviness $W_{cz}$, eccentricity $e_{cz}$, etc.

The control vector $\vec{U}$, which is formed in the main-motion drives, is passed to the TS of feeds and additional motions, allowing to move and (or) position in place the actuators and measurement systems. In machine tools, the control vector is shaped by the main-motion electric drive, which allows part $n_{cz}$ to rotate, and by feed drives, which, in a general case, are used to move the part along three coordinates $V_{x}$, $V_{y}$, and $V_{z}$. Special drives also make it possible to control the angle of rotation $\vartheta_{i}$ of machine tool actuators and measuring systems about different axes:

(2) $$\vec{U}=\left(n_{cz},V_{x},V_{y},V_{z},\vartheta_{i},\ldots \right)$$

The TS of cutting machine tools transforms the input (control signals) into displacements and displacement velocities of TS components, thus defining the technological variables $\vec{T}$ that determine the course of the TP. The components of vector $\vec{T}$ include: cutting speed $v_{c}$ depth of cut $a_{p}$, cross-feed rate $v_{f}$ and the dimension of static set-up ${∆}_{s}$:

(3) $$\vec{T}=\left(v_{c},a_{p},\;v_{f},{∆}_{s},\ldots \right)$$

The TP of machining involves the removal of material from the workpiece, which leads to the formation of a cutting force vector ${\vec{F}}_{sk}$. The force processes in the cutting zone can be characterized using parameters such as cutting force $F_{sk}$, cutting power $P_{sk}$, moment of the spindle shaft $M_{wr}$, and moment of the feed drive $M_{f}$. The TS of the cutting machine tool transforms control signals into displacements and displacement velocities:

(4) $${\vec{Y}}_{pt1}=\left({\vec{F}}_{sk},P_{sk},\;M_{wr},M_{f},\ldots \right)$$

The output variables of a TP can also include efficiency $W_{PT}$, material removal rate $V_{m}$, cutting tool wear rate $V_{z}$, various economic indices $E_{i}$, and others:

(5) $${\vec{Y}}_{pt2}=\left(W_{PT},\;V_{m},V_{z},E_{i},\ldots \right)$$

To increase the accuracy of machining of parts, especially low-rigidity parts, TS, apart from providing control from feed drives and main-motion drives, can use special control devices which change the elastic-deformable state of parts by applying forces to them. These forces include clamping force $F_{x1}$, non-axial tensile force, which also produces bending moment $M_{zg}=F_{x1}\times e$ (*e*—eccentricity of the tensile force); one or more additional counter-forces $F_{d}$; bending moments $F_{zg}$; electromagnetic forces $F_{em}$, and others. The application of additional forces is described in the schematic (Figure 1) by the following vector:

(6) $${\vec{F}}_{st}=\left(F_{st},\;F_{d},e,M_{zg},F_{em}\ldots \right)$$

A TS is affected by many disruptions (interfering factors), which primarily include variations in force variables caused by the implementation of the technological process. These disruptions can be considered as internal system disruptions.

The accuracy of mechanical devices (in particular, precision cutting machine tools, optical-mechanical measuring systems, measuring machines, and robots) is also significantly influenced by elastic deformations generated by the kinematic effect of the machine foundation.

In general, the effect of the foundation can be represented as a vector of kinematic effects ${\vec{R}}_{k}$ causing additional relative displacement of actuators (end components), such as the cutter and the part, in the case of a machine tool, or components of an optical measuring system. In order to reduce vibration coming from the vibration-active machine foundation, special elastic elements are often placed between the foundation and the load-bearing elements of the technological system, which act as shock absorbers. The kinematic forces acting on the load-bearing elements of the technological system (vector ${\vec{R}}_{o}$), which cause the displacement of its actuators (end elements), depend not only on the properties of the TS, but also on the characteristics of the shock absorbers or, when an active anti-vibration protection system is used, on vector of control ${\vec{F}}_{o}$ of anti-vibration system actuators.

A TS is also affected by disruptions such as temperature changes T, variability of the friction coefficient $f_{tar}$, clearances α, wear of components $J_{i}$, etc. These additional disruptions can be described by the following vector:

(7) $${\vec{B}}_{1}=\left(T,f_{tar},\alpha ,J_{i},\ldots \right)$$

A disruption of a technological process can be represented as a vector ${\vec{B}}_{2}$ consisting of the following components: a change in length allowance $b_{l}$ and radius allowance $b_{r}$ of the part, a change in the hardness q of the part, a change in the cutting properties $r_{i}$ of the tool, etc.:

(8) $${\vec{B}}_{2}=\left(b_{l},b_{r},q,r_{i},\ldots \right)$$

The general structure of a system of technological devices can be tailored to take into account the specific properties of the control object. In the case of cutting machine tools, these properties include the earlier-discussed set of disruptions and relationships between variables. 

The most important goals of controlling machining systems are to intensify the technological process and increase the machining accuracy.

## 3. Model of a Machining System for the Processing of Low-Rigidity Parts

By controlling the technological process of machining of axisymmetric low-rigidity parts, one can increase the accuracy of the shape and dimensions of those parts, improve the technical and economic indices of machining, and increase the operational reliability of the machining system. It is, therefore, important to develop a mathematical description of a machining system. 

**Figure 2:**
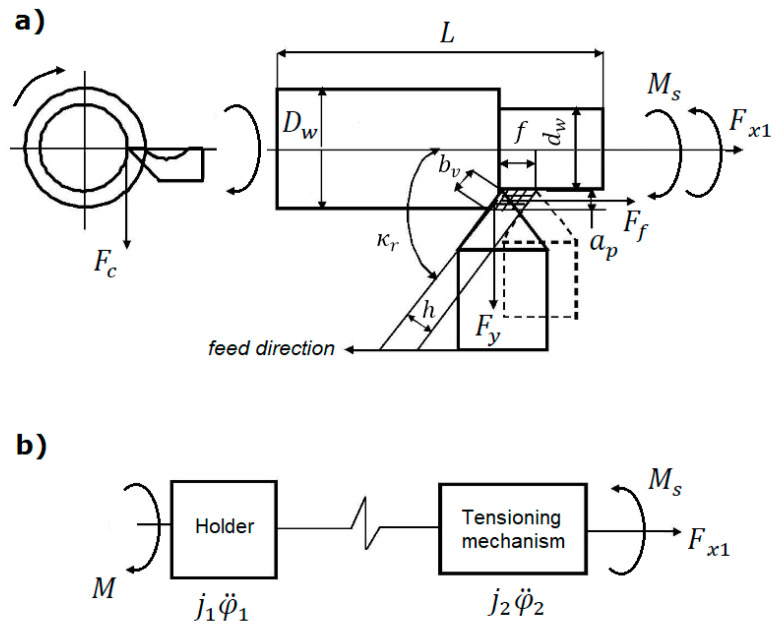
Calculation scheme of the machining system (**a**), model of the machining system (**b**).

Modelling should take into account the relationships among parameters, which allow to achieve the required machining accuracy and appropriate control of the TP. The effectiveness of solving such problems largely depends on the choice of goal and modelling methods. In the analyzed case, the aim is to determine feasible parameters for the control of the process of turning of low-rigidity parts, based on the model of an elastic system. To achieve this goal, we developed a structural and a calculation scheme and a model of controlling the machining system (Figure 2).

Based on Figure 2 and the second-order Lagrange equation, we developed a mathematical model of the machining system for the turning of low-rigidity parts:

(9) $$\frac{d}{dt}\left(\frac{\partial T}{\partial {\dot{\varphi }}_{i}}\right)-\frac{\partial T}{\partial \varphi_{i}}+\frac{\partial \phi }{\partial {\dot{\varphi }}_{i}}+\frac{\partial P}{\partial \varphi_{i}}=Q_{i}$$

where: 

T,P—kinetic and potential energies of the system, respectively;

ϕ—Rayleigh dissipation function;

${\dot{\varphi }}_{i},\;\varphi_{i}$—generalized coordinate and generalized velocity of the system’s model;

$Q_{i}$—generalized forces.

The kinetic and potential energies and the Rayleigh dissipation function of the system under consideration are calculated as follows:

(10) $$T=\frac{1}{2}\left(j_{1}{\dot{\varphi }}_{1}^{2}+j_{2}\varphi_{2}^{2}\right),\;P=\frac{1}{2}c{\left(\varphi_{1}+\varphi_{2}\right)}^{2},\;\emptyset =\frac{1}{2}b{\left({\dot{\varphi }}_{1}-{\dot{\varphi }}_{2}\right)}^{2}$$

The components of the Lagrange equations are defined as partial derivatives of displacement, velocity, time, and generalized force:

(11) $$\begin{array}{c} \frac{P}{\partial \varphi }=c\left(\varphi_{1}-\varphi_{2}\right),\;\frac{\partial T}{\partial {\dot{\varphi }}_{i}}=j_{i}\dot{\varphi_{i}},\;\frac{\partial \emptyset }{\partial \dot{\varphi }}=b\left({\dot{\varphi }}_{1}-{\dot{\varphi }}_{2}\right),\;\frac{d}{dT}\left(\frac{\partial T}{\partial {\dot{\varphi }}_{i}}\right)=j_{i}{\ddot{\varphi }}_{i}\\ Q_{1}=M_{n},\;Q_{2}=M_{0} \end{array}$$

where:

$j_{1},j_{2}$—moments of inertia of the rotating masses of the system;

${\dot{\varphi }}_{1}$,$\;{\dot{\varphi }}_{2}$—angular velocities in the machining of the active and passive sections of the shaft;

$\varphi_{1},\;\varphi_{2}\;$—angular displacements;

$c=c_{k}+c_{l}$—total stiffness (angular and linear) of the shaft;

c*—*ductility factor of the shaft;

$M_{n},M_{0}$—the system’s driving torque and frictional torque.

Once the components of the Lagrange equations have been determined, a mathematical model of the machining system can be formulated in the following way, taking into account the driving and the frictional torques:

(12) $$\{\begin{array}{c} j_{1}{\ddot{\varphi }}_{1}=M_{n}-b\left({\dot{\varphi }}_{1}-{\dot{\varphi }}_{2}\right)-c\left(\varphi_{1}-\varphi_{2}\right)\\ j_{2}{\ddot{\varphi }}_{2}=b\left({\dot{\varphi }}_{1}-{\dot{\varphi }}_{2}\right)-c\left(\varphi_{1}-\varphi_{2}\right)-M_{0} \end{array}$$

Models of elastic curves of low-rigidity parts machined in the elastic-deformable state take into account the bending moments relative to the *X* axis and elastic deformations relative to this axis, which have the largest impact on shape errors in the longitudinal direction. Component $F_{c}$ of the cutting force greatly affects the torsion of the part, and its bending effect can be represented as a load:

(13) $$F_{sg}=\sqrt{F_{c}{}^{2}+F_{p}{}^{2}}$$

The values of the cutting force components $F_{c}$, $F_{p}$, and $F_{f}$ are defined by technological conditions, such as machining parameters and geometry [31].

The value of force *F_c_* acting in the direction of the cutting speed can be roughly defined as:

(14) $$F_{c}=\sigma \times A_{p}$$

Hence, the value of torque is given by:

(15) $$M_{skr}=r_{w}\times F_{c}$$

where:

σ=0.35×q—material’s ultimate strength;

q*—*material’s Brinell hardness;

$A_{p}=h\times b$—surface area of the machined layer;

$h=f\sin\left(\kappa_{r}\right)$—machining layer thickness;

$b=\frac{a_{p}}{\sin\left(\kappa_{r}\right)}$—width of the machined layer ($\kappa_{r}=45^{{}^{\circ}}$—cutting edge angle);

$f=\frac{v_{c}}{n}$—feed,$\;v_{c}=\frac{\pi \cdot D\cdot n}{1000}$—cutting speed;

n—rotational frequency;

D—shaft diameter;

$a_{p}$—depth of cut; $r_{w}$—shaft radius.

During machining, the depth of cut is reduced to the value $a_{p\;fak}=a_{p\;okr}-y_{0}$, which can be calculated using the following equation:

(16) $$a_{p\;fak}=\frac{F_{p}}{c}=\frac{0.5\times F_{c}}{c}$$

The total stiffness and ductility of the shaft is determined as:

(17) $$c=c_{k}+c_{l}=\frac{GJ_{p}}{l}+\frac{EJ_{x}}{l},\;k_{c}=\frac{0.064\times c}{\omega }$$

where:

$c_{k},c_{l}$—angular and linear stiffness of the shaft;

$k_{c}\;$—shaft ductility factor; l—shaft length;

G,E—shear modulus and Young’s modulus of the material;

$J_{p},J_{x}$—polar and axial moments of inertia;

$\omega =\sqrt{\frac{c}{J_{i}}}$—process frequency.

Relative longitudinal deformation of the shaft in tension:

(18) $$\varepsilon =\frac{∆l}{l}$$

Absolute elongation of the shaft in tension:

(19) $$∆l=\frac{F_{x1}\times l}{EF}$$

hence, the allowable value of the cutting force is:

(20) $$F_{x1d}=\left[\sigma_{r}\right]\times S$$

where:

$\sigma_{r}$—allowable stress in tension; 

S—cross-sectional area of the shaft.

Taking into account the tensile force and the absolute elongation of the shaft in tension, one can determine the relative longitudinal deformations using Hooke’s law:

(21) $$\varepsilon =\frac{\left[\sigma_{p}\right]}{E}$$

and the design value of tensile force in this case is $F_{x1}=c\times ∆l$.

To develop a control strategy for a dynamical system, it is necessary to use the system of Equation (12), taking into account the following assumptions:

(1) The rotor of the TS’s motor moves in accordance with a specific principle $\varphi_{0}\left(t\right)$, which corresponds to the ideal motor performance;
(2) Reduced moment of inertia $j_{1}\left(\varphi_{1}\right)$, is constant;
(3) The modulus of reduced technological frictional (drag) torque produced by frictional forces generated by the cutter changes according to the equation: $M_{p}=M_{s}+M_{0}\sin\omega t$, ($M_{0}$—amplitude of cutter vibrations relative to the mean value).

When motor power is sufficiently high, it can be assumed that the motion of its rotor $\varphi_{1}\left(t\right)$ is independent of changes in frictional torque $M_{p}$ and moment of inertia $j_{2}$. When the dependence $\varphi_{1}\left(t\right)$ is given, the Equation (12) can be written as:

(22) $$j_{2}\varphi +b\dot{\varphi }+c\ddot{\varphi }=M_{p}+J_{1}\ddot{\varphi_{1}}$$

where: $\varphi =\varphi_{1}-\varphi_{2}$—angular displacements of the shaft.

Assuming that angular velocity ${\dot{\varphi }}_{1}$ is constant, the equation of motion of the shaft can be formulated as follows:

(23) $$j_{2}\ddot{\varphi }+b\varphi +c\dot{\varphi }=M_{p}$$

The first Equation (12) in this case can only be used to determine the driving torque which allows the rotor to move in the required way. The motion of the shaft with a reduced moment of inertia $j_{2}$ can therefore be considered as consisting of a main motion ${\dot{\varphi }}_{1}$ and an additional motion with velocity ${\dot{\varphi }}_{c1}$, which usually is a vibrating motion. 

## 4. Mathematical Modelling and Evaluation of the Energy State of a Dynamical System

One of the primary tasks is to determine the energy required to form a part in the actual forming process. This is expressed as power correction of system elements:

(24) $$\left|P_{z}-P_{o}\right|\leq \varepsilon$$

where:

$P_{z}$—cutting power; 

$P_{o}$—design value of cutting power, ∀ ε (0< ε<1)*—*a low value.

Power analysis makes it possible to evaluate the energy state of the process of forming a part [32]. Mathematical models of the behavior of a dynamical system in the process of forming a low-rigidity shaft are presented in Section 3. They are described using the second-order Lagrange equation. The energy of these motions, on the other hand, is determined using the Hamilton equations.

The second-order Lagrange dynamic Equation (9) can be transformed into canonical equations. The equations form a system of n second-order equations with respect to n functions $q_{i}\left(t\right)$. The order of this system is 2n. To reduce Equation (9) to canonical form, Lagrange variables $q_{i}$ and ${\dot{q}}_{i}$ are replaced with the canonical variables $q_{i}$ and $p_{i}$ (force coordinates and force impulses, respectively).

The transform of function $L=\left(q_{i},{\dot{q}}_{i},\;t\right)$ with respect to variables $q_{i}$ is a Hamilton function for systems operating in a potential force field [19]:

(25) $$H\left(q_{i},p_{i},\;t\right)=\overset{n}{\underset{i=1}{\sum }}p_{i}q_{i}-L\left(q_{i},{\dot{q}}_{i},t\right)$$

$q_{i}$ can be expressed as $q_{i},p_{i},t.$

(26) $$p_{i}=\frac{\partial L}{\partial {\dot{q}}_{i}}=\frac{\partial \left(T+n\right)}{\partial {\dot{q}}_{i}}\left(i=1,\;2,\ldots ,\;n\right)$$

In the case under consideration, apart from potential forces, the system is also affected by non-potential forces. When canonical variables are introduced, Equation (26) can be written as

(27) $$\frac{dq_{i}}{dt}=\frac{\partial H}{\partial P_{i}},\frac{dP_{i}}{dt}=-\frac{\partial H}{\partial q_{i}}+Q_{i}$$

expression (27) is a Hamilton equation when the system is non-conservative.

The full-time derivative of the Hamilton function is defined using Equation (26) as follows:

(28) $$\frac{dH}{dt}=\frac{\partial H}{\partial t}+\sum_{i=1}^{n}\frac{\partial H}{\partial q_{i}}{\dot{q}}_{i}+\sum_{i=1}^{n}\frac{\partial H}{\partial P_{i}}\dot{P}\;\left(i=1,\;3\right)$$

Thus, the motion of a dynamical system can be represented by the Hamilton Equations [15]. It follows from Equation (28) [12] that in the case of a dynamical system $\left({\dot{q}}_{i}={\dot{\varphi }}_{i}\right)$

(29) $$\{\begin{array}{c} \frac{\partial H}{\partial \varphi_{d}}=-\frac{\partial T}{\partial \varphi_{d}}=0;\;P_{d}=\frac{\partial T}{\partial \varphi_{d}}=j_{d}{\dot{\varphi }}_{d}\\ \frac{\partial H}{\partial \varphi_{0}}=-\frac{\partial T}{\partial \varphi_{0}}=0;\;P_{0}=\frac{\partial T}{\partial \varphi_{p}}=j_{0}{\dot{\varphi }}_{0} \end{array}$$

Taking into account the invariability of the system’s parameters, this can be written as:

(30) $$\frac{\partial H}{\partial t}=-\frac{\partial T}{\partial t}=0$$

Given Equations (27) and (29), we obtain a system of canonical equations:

(31) $$\{\begin{array}{c} \frac{d\varphi_{d}}{dt}=-\frac{\partial H}{\partial P_{d}}=j_{D}^{-1}P_{d};\;\frac{dP_{d}}{dt}=\frac{\partial H}{\partial \varphi_{d}}+Q_{d}\\ \frac{d\varphi_{0}}{dt}=-\frac{\partial H}{\partial P_{0}}=j_{B}^{-1}P_{o};\;\frac{dP_{0}}{dt}=\frac{\partial H}{\partial \varphi_{0}}+Q_{0} \end{array}$$

After substituting Equations (29) and (31) into (28), we obtain:

(32) $$\frac{dH}{\partial t}=\frac{\partial H}{\partial t}-\overset{n}{\underset{i=1}{\sum }}\frac{\partial T}{\partial \varphi_{i}}+\overset{n}{\underset{i=1}{\sum }}\frac{\partial H}{\partial P_{i}}\left(-\frac{\partial H}{\partial \varphi_{i}}+Q_{i}\right)=\overset{n}{\underset{i=1}{\sum }}\left(-\frac{\partial H}{\partial \varphi_{i}}+Q_{i}\right){\dot{\varphi }}_{i}$$

The following equation is derived from Equation (32):

(33) $$\frac{dH}{dt}=\overset{n}{\underset{i=1}{\sum }}\left(-\frac{\partial H}{\partial \varphi_{i}}+Q_{i}\right){\dot{\varphi }}_{i}=\overset{n}{\underset{i=1}{\sum }}Q_{i}{\dot{\varphi }}_{i}$$

Let the system, as a stationary object with constant parameters and a given cutting power, have the general coordinate of constraints $\varphi_{w}$. By substituting Equation (33) into (31), we obtain:

(34) $$P_{\varepsilon }=\overset{n}{\underset{i=1}{\sum }}\left[\left(-\frac{\partial H}{\partial \varphi_{i}}+Q_{3}\right)-\left(-\frac{\partial H}{\partial \varphi_{i}}+Q_{0}\right)\right]{\dot{\varphi }}_{i}=\overset{n}{\underset{i=1}{\sum }}\left(Q_{3}-Q_{0}\right){\dot{\varphi }}_{i}$$

The correction of the energy state is determined by condition [20]:

(35) $$|Q_{3}-Q_{0}|=\{\begin{array}{c} 0,\;\text{if the respective functioning parameters are equal},\\ \neq 0\;\text{otherwise}. \end{array}$$

**Figure 3:**
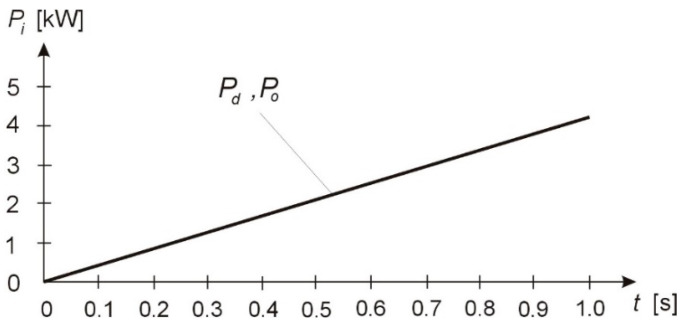
Power curve for the TP.

By substituting the values of angular velocity ${\dot{\varphi }}_{i}$ and driving and frictional torques $Q_{i}=M_{i}=j_{i}{\ddot{\varphi }}_{i}$ of Equation (34) into (35), one can determine the energy state of the system for the machining of low-rigidity shafts; this energy state corresponds to conditions defined by Equations (24) and (35). The values and curves of the energy state of the dynamical system during the machining process are shown in Table 1 and Figure 3.

## 5. Control of the System for the Machining of Axisymmetric Low-Rigidity Parts

Machining systems are complex multi-component systems that execute mechanical, electrical, and other energy-related processes. In the system analyzed in this study, an energy exchange occurs during machining, between the force that puts and keeps the shaft in motion and the cutting force. Given this, it is necessary to determine the amount of energy consumed. The process is dynamic, because such a system can be described using [32].

Rotor output torque:

(36) $$j\ddot{\varphi }=M_{n}=Ri_{w}$$

Change of voltage in the rotor circuit:

(37) $$U=L\frac{di_{w}}{dt}Ri_{w}$$

where: 

$M_{n}$—driving torque; 

L*—*inductance; 

$i_{w}$—rotor current ($i=\frac{dq_{l}}{dt}$, $q_{l}\;$*—*electric charge); 

R*—*structural constant (drag); 

U—voltage.

The energy exchange equation, in the case of the system under consideration Equation (36), when frictional torque is generated, will take the following form:

(38) $$j\ddot{\varphi }=M_{n}-M_{op}=R\left(i_{w}-i_{op}\right)=Ri_{e}$$

where:

$M_{op}$—frictional torque; 

$i_{op}$—current, aka (cutting) force; 

$i_{e}$—equivalent current corresponding to the dynamic process of machining a shaft.

When the value of inductance is sufficiently low, it is assumed that *L =* 0 [8,10], and hence the electromotive force of the motor is:

(39) $$E_{SEM}=R\left(i_{w}-i_{op}\right)$$

Based on the above, it is possible to determine the system’s energy exchange power:

(40) $$P_{op}=R{\left(i_{w}-i_{op}\right)}^{2}$$

We investigated how this process is executed when machining a shaft. Analogously to Equation (40), in the case of a mechanical process [8,11]:

(41) $$P_{op}=R{\left(i_{w}-i_{c}\right)}^{2}=b{\left({\dot{\varphi }}_{i}-{\dot{\varphi }}_{r}\right)}^{2}$$

where: 

b—shaft ductility factor; 

${\dot{\varphi }}_{1}$—angular speed of the active part of the processed shaft; 

${\dot{\varphi }}_{2}$—angular speed of the passive part of the processed shaft; 

${\dot{\varphi }}_{r}={\dot{\varphi }}_{1}-{\dot{\varphi }}_{2}$—difference in the angular velocities of the active and passive shaft sections produced by the frictional torque.

To obtain optimal control at R *= const* and b *= const* it is assumed that: $u={\dot{\varphi }}_{1}-{\dot{\varphi }}_{2}={\dot{\varphi }}_{r}$, ${\dot{\varphi }}_{i}=\frac{d\varphi_{i}}{dt}$. Energy losses in the process of machining a shaft are determined by minimizing the function [33] until a given time *T*:

(42) $$J_{\left(u\right)}=\int_{0}^{T}u^{2}dt$$

subject to the following conditions:

(43) $$\varphi =y_{1},\;\dot{\varphi }=y_{2},\;{\dot{y}}_{2}\frac{1}{j_{2}}\left(by_{2}+cy_{1}\right)-u_{p}-u_{0}sin\omega t$$

(44) $$\varphi_{i}\left(0\right)=\varphi_{0}\left(0\right),\;{\dot{\varphi }}_{i}\left(0\right)={\dot{\varphi }}_{0}\left(0\right),\varphi_{i}\left(t\right)=\varphi_{0}\left(t\right),\;{\dot{\varphi }}_{i}={\dot{\varphi }}_{0}\left(t\right),\;i=\bar{1,\;n},\;0\leq t\leq T$$

The Hamilton–Pontryagin function for a dynamical system can be written as:

(45) $$H\left(\varphi ,u,t,\Psi_{i},\Psi_{0}\;\right)=-\Psi_{0}u^{2}+y_{2}\Psi_{1}+\Psi_{2}{\dot{y}}_{2}$$

and a coupled system can be written as:

(46) $$\frac{d\Psi_{1}}{dt}=-\frac{\partial H}{\partial y_{1}}=-j_{1}{}^{-1}c\Psi_{2},\;\frac{d\Psi_{2}}{dt}=-\frac{\partial H}{\partial y_{2}}=-\Psi_{1}+j_{1}{}^{-1}c\Psi_{2}$$

with a constraint on control |u|≤1.

For the problem in question to be solved, the following necessary condition must be satisfied

(47) $$H\left(q_{i}\left(t\right),\;u\left(t\right),t,\Psi_{i},\Psi_{0}\right)=\underset{u\in U}{\max}\;H\left(q_{i}\left(t\right),u,t,\Psi_{i}\left(t\right),\Psi_{0}\right)$$

It follows from conditions $\underset{\left|u\right|\leq 1}{\max}\;H$ that $u=\frac{\Psi_{2}}{2}$ when $\Psi_{2}\neq 0$, because the coupled system in Equation (46) is homogeneous with respect to $\Psi_{i}$ and constant

(48) $$\Psi_{0}\left(t\right)=-1,\;0\leq t\leq T$$

can take any value.

The system described by Equations (42)–(48) was studied using the Runge–Kutta numerical methods. 

To determine the auxiliary functions, the coupled system described by Equation (46) was tested numerically when changing the design parameters $b_{i}$, $c_{i}$, $j_{i}$ within the following ranges: b = 0.3–15 N⋅m⋅s/rad; c = 30,000–60,000 N⋅m/rad; j_2_ = 0.08–0.75 N⋅m⋅s^2^.

An evaluation of the solutions of system described by Equation (46) shows that changes in the moments of inertia and elastic-dissipative forces significantly alter the function of the variables $\psi_{1}$, ${\dot{\varphi }}_{1}$, $\psi_{2}$, ${\dot{\varphi }}_{2}$, i.e., motion of the shaft. To be able to increase the accuracy of the shape and dimensions of shafts, one has to determine the variables of the coupled system which ensure normal functioning of the dynamical system [34].

**Figure 4:**
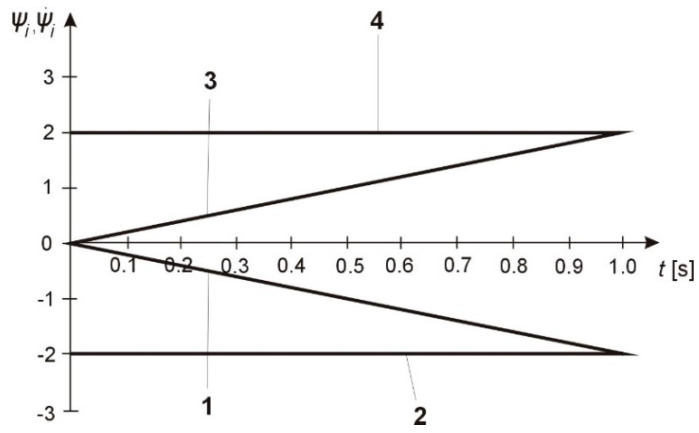
Graph of changes of auxiliary functions: $1-\psi_{1}$, $\psi_{2};$ $2\;–{\dot{\psi }}_{1}$,$\;{\dot{\psi }}_{2}$ for u(t)=−1; $3-\psi_{1}$, $\psi_{2}$, $4-\dot{\psi_{1}}$,$\;\dot{\psi_{21}}$ for u(t)=+1.

Parameters $b_{i}$, $c_{i}$, and $j_{i}$ of the solution for the coupled system shown in Table 2 and in Figure 4 provide a basis for the analysis of the transition processes of the shaft. 

By substituting the values of moments of inertia and elastic-dissipative forces Equation (43), one can solve the boundary-value problem defined by the maximum principle.

**Figure 5:**
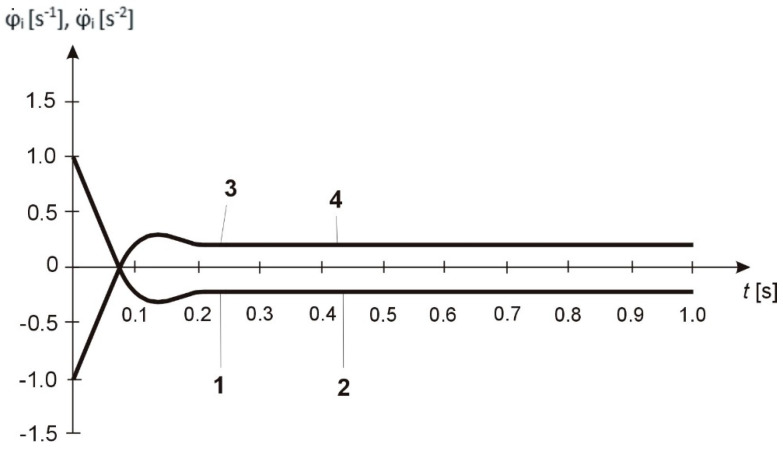
Graph of changes in the function of angular velocities—1, 3 and angular accelerations—2, 4 of the shaft for: 1, 2—u(t)=+1; 3, 4—u(t)=−1.

Boundary-value relationships for shaft velocities and accelerations during the transition process and the maximum values of *H*-functions were obtained (Table 3, Figure 5).

The effect of moments of inertia and elastic-dissipative forces on the nature of and changes in the motion of the shaft were investigated. A change in the moment of inertia clearly affects the angular velocities and accelerations of the shaft. To reduce the range of these changes, the coefficients of stiffness and ductility of the shaft were adjusted. Stiffness significantly affects the range of angular vibrations of the shaft. Increasing the stiffness leads to a reduction in shaft deformation and a shorter transition process. By increasing the ductility coefficient, on the other hand, one can significantly reduce the amplitude of vibrations of the angular velocities of the shaft being machined. 

The amplitude and frequency of vibrations of angular velocities and accelerations of the shaft depend on the moment of inertia and elastic-dissipative forces. The solutions to Equations (42)–(48) defined by the maximum principle, correspond to the condition maximizing the scalar product *H_i_* of the Hamilton–Pontryagin function describing the process of machining the shaft. 

The results obtained in this way allow to study the dynamical system which is under the influence of moments of cutting forces. 

To determine the optimal values of the driving forces and design parameters of the dynamical system, the driving torque and the moments of cutting forces were varied. It was established that the uniformity of shaft motion depends on the value of the moments of cutting forces. A change in the moments of cutting forces results in a deviation from the specified angular shaft velocity and duration of the transition process. This means that an increase in the moments of cutting forces, when shaft machining parameters are given/other parameters being equal, significantly affects the expected machining accuracy. Given the values of the moments of inertia of the rotating masses and the coefficients of stiffness and ductility of the shaft, we found the values of the driving torque and the moments of cutting forces.

**Figure 6:**
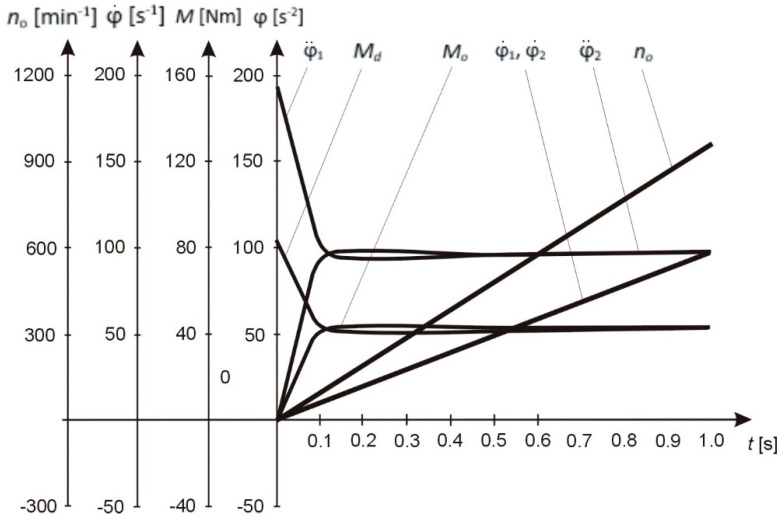
The nature of changes in the parameters of the technological process.

The results of numerical calculations of the system described by Equatin (12) shown in Table 4 and Figure 6 can be used to determine the optimal geometric, design, and functional parameters of the shaft:

(1) Geometrical dimensions of the shaft: L = 600 mm; d = 40 mm; r_w_ = 20 mm;
(2) Machining conditions: cutting speed—v_c_ = 115.552 m/min; feed—f = 0.125mm/rev; machining time—t = 5.12 min; depth of cut—a_p_ = 0.1 mm; width of the machined layer—b = 0.88 mm; thickness of the machined layer—h = 0.14 mm; rotational speed of the shaft (number of turns)—n = 920 rev/min;
(3) Structural characteristics of the shaft: stiffness coefficient—c = 45573.3 N⋅m/rad; ductility coefficient—b = 9.2 N⋅m⋅s/rad; moment of inertia of machine tool holder—j_1_ = 0.45 N⋅m⋅s^2^; moment of inertia of tensioning mechanism—j_2_ = 0.45 N⋅m⋅s^2^; cross-sectional area of the shaft being machined—S = 0.001256 m^2^; elastic modulus—E; = 2.1 × 10^5^ MPa; shear modulus G = 8.1 × 10^4^ MPa; allowable stress in tension—σ_r_ = 90 MPa; ultimate strength—σ = 0.35⋅HB; material’s Brinell hardness—HB 250; longitudinal strain in tension—ε = 0.00416; absolute elongation of the shaft in tension—Δl = 0.0025 m; material—S235 steel;
(4) Operation parameters of the dynamical system: driving torque—M_n_ = 43.35 N⋅m; force acting in the direction of cutting speed—F_z_ = 10.99 N; components of the cutting force—F_y_ = 5.4 N, F_x_ = 3.3 N; allowable force in tension—F_dop_ = 11.304 N; design tensile force—F_r_ = 114.4 N; elastic deformations of the shaft—y_0_ = 1.2 × 10^4^ m; elastic deformations of the cutting tool—y_s_ = 3 × 10^5^ m, obtained analogously to the calculations for the shaft with c_s_ = 1.62 × 10^6^ N/m, b_s_ = 122.2 N s/m.

In this way, a mathematical model of the functioning of the system was obtained, which allows to optimally control the dynamical system during the machining of parts. The proposed method for modelling the TP of machining low-rigidity shafts in which the operation parameters of the technological system are interconnected by mutual relationships, permits to control the system under specific conditions and obtain a higher shape accuracy.

The results of the experiments show that the proposed mathematical models describe the properties of the original object with sufficient accuracy. 

## 6. Conclusions

In this study, we analyzed the qualitative relationships among TS variables, such as elastic deformations of the TS of the cutting machine tool which cause changes in the spatial position of the cutter and the part, reducing machining accuracy. We also presented a schematic of a machining system for the processing of low-rigidity parts. Conducted research helped to:

(1) Define important variables directly characterizing the accuracy and quality of machining, e.g., diameter of the part being machined, the roughness and waviness of its surface, and eccentricity.
(2) Determine control signals from the main-motion and feed drives and additional forces generated by special control devices, which change the elastic-deformable state of the part, such as: clamping force, non-axial tensile force (which also produces bending moment), bending moments, and others.
(3) Develop a structural scheme and a calculation scheme, a control model, and a mathematical model using the second-order Lagrange equation of the machining system for the turning of low-rigidity parts and presented equations that allow to determine the energy state of this system.
(4) Obtain a mathematical model of the functioning of the system, which allows to optimally control the system during the machining of parts—the proposed method for modelling the TP of machining of low-rigidity parts in which the operation parameters of the TS are interconnected by mutual relationships, allows to control the system under specific conditions and achieve a higher shape accuracy.
(5) Based on the results of numerical calculations, it is possible to determine the optimal values of the geometric, design, and functional parameters of the shaft being machined.

The experimental results confirm that the proposed mathematical models describe the properties of the original object with sufficient accuracy. To extend our research in the future, we intend to conduct validation of proposed methods with the use of axisymmetric low-rigidity shafts made of different types of steel. Important aspect of further research states the need of proposed solution verification in the real machining processes (in the production environment). Moreover, research should be continued in the aspect of another machining parameters identification and inclusion.

## 7. Patents

(1) Lathe tailstock [patent no. 212961]/Victor Taranenko, Antoni Świć, Dariusz Wołos, Gieorgij Taranenko; author: Victor Taranenko, Antoni Świć, Dariusz Wołos, Gieorgij Taranenko.—Patent no.; Patent application no.//Official Gazette of the Patent Office, 2012, No. 12, p. 2897
(2) Lathe tailstock [patent no. 211537]/Victor Taranenko, Antoni Świć, Dariusz Wołos, Gieorgij Taranenko; author: Victor Taranenko, Antoni Świć, Dariusz Wołos, Gieorgij Taranenko.—Patent no.; Patent application no.//Official Gazette of the Patent Office, 2012, No. 5, p. 1073
(3) Lathe tailstock [patent no. 213606]/Victor Taranenko, Antoni Świć, Dariusz Wołos, Gieorgij Taranenko, Jakub Szabelski; author: Victor Taranenko, Antoni Świć, Dariusz Wołos, Gieorgij Taranenko, Jakub Szabelski.—Patent no.; Patent application no.//Official Gazette of the Patent Office, 2013, No. 4, p. 851
(4) Lathe tailstock [patent no. 213607]/Victor Taranenko, Antoni Świć, Igor Bagimov, Gieorgij Taranenko, Jakub Szabelski; author: Victor Taranenko, Antoni Świć, Igor Bagimov, Gieorgij Taranenko, Jakub Szabelski.—Patent no.; Patent application no.//Official Gazette of the Patent Office, 2013, No. 4, p. 851
(5) Lathe tailstock [patent no. 213608]/Victor Taranenko, Antoni Świć, Igor Bagimov, Gieorgij Taranenko, Jakub Szabelski; author: Victor Taranenko, Antoni Świć, Igor Bagimov, Gieorgij Taranenko, Jakub Szabelski.—Patent no.; Patent application no.//Official Gazette of the Patent Office, 2013, No. 4, p. 851
(6) Lathe tailstock [patent no. 214058]/Victor Taranenko, Antoni Świć, Dariusz Wołos, Gieorgij Taranenko, Jakub Szabelski; author: Victor Taranenko, Antoni Świć, Dariusz Wołos, Gieorgij Taranenko, Jakub Szabelski.—Patent no.; Patent application no.//Official Gazette of the Patent Office, 2013, No. 6, p. 1426

## Figures and Tables

**Table 1:** Power consumed by the machine tool motor during cutting.

*T* [s]	0	0.1	0.2	0.3	0.4	0.5	0.6	0.7	0.8	0.9	1
*P*_d_ [kW]	0	0.44	0.84	1.25	1.67	2.08	2.5	2.9	3.34	3.76	4.17
*P*_o_ [kW]	0	0.38	0.84	1.25	1.67	2.08	2.5	2.9	3.34	3.76	4.17
*ε*	0	0.06	0	0	0	0	0	0	0	0	0

**Table 2:** Values of auxiliary functions.

*T* [s]	$\Psi_{1}$	${\dot{\Psi }}_{1}$	$\Psi_{2}$	${\dot{\Psi }}_{2}$	$\Psi_{1}$	${\dot{\Psi }}_{1}$	$\Psi_{2}$	${\dot{\Psi }}_{2}$
1	2	3	4	5	6	7	8	9
-	-	*u = +*1	-	-	-	*u = –*1	-	-
0	0	−2	0	−2	0	2	0	2
0.1	−0.2	−2	−0.2	−2	0.2	2	0.2	2
0.2	−0.4	−2	−0.4	−2	0.4	2	0.4	2
0.3	−0.6	−2	−0.6	−2	0.6	2	0.6	2
0.4	−0.8	−2	−0.8	−2	0.8	2	0.8	2
0.5	−1	−2	−1	−2	1	2	1	2
0.6	−1.2	−2	−1.2	−2	1.2	2	1.2	2
0.7	−1.4	−2	−1.4	−2	1.4	2	1.4	2
0.8	−1.6	−2	−1.6	−2	1.6	2	1.6	2
0.9	−1.8	−2	−1.8	−2	1.8	2	1.8	2
1	−2	−2	−2	−2	2	2	2	2

**Table 3:** Angular velocities, accelerations, and *H*-functions of the machined shaft.

*t* [s]	$\dot{\varphi }\;[s^{-1}]$	$\ddot{\varphi }\;[s^{-2}]$	*H*_1_	$\dot{\varphi }[s^{-1}]$	$\ddot{\varphi }\;[s^{-2}]$	*H*_1_
	*u* = +1			*u = −*1		
0	0	1	−1	0	−1	−1
0.1	0.0002	−0.2	−1	−0.0002	0.2	−1
0.2	0.00001	−0.2	−1	−0.00001	0.2	−1
0.3	0.00001	−0.2	−1	−0.00001	0.2	−1
0.4	0.00001	−0.2	−1	−0.00001	0.2	−1
0.5	0.00001	−0.2	−1	−0.00001	0.2	−1
0.6	0.00001	−0.2	−1	−0.00001	0.2	−1
0.7	0.00001	−0.2	−1	−0.00001	0.2	−1
0.8	0.00001	−0.2	−1	−0.00001	0.2	−1
0.9	0.00001	−0.2	−1	−0.00001	0.2	−1
1	0.00001	−0.2	−1	−0.00001	0.2	−1

**Table 4:** Values of parameters of the technological process of shaft machining.

*t* [s]	${\dot{\varphi }}_{1}\;[s^{-2}]$	${\ddot{\varphi }}_{1}\;[s^{-2}]$	$M_{d}\;[\mathrm{Nm}]$	${\dot{\varphi }}_{2}\;[s^{-2}]$	$\;{\ddot{\varphi }}_{2}\;[s^{-2}]$	$M_{o}\;[\mathrm{Nm}]$	$n_{o}\;[\min^{-1}]$
0	0	193.17	86.93	0	−0.49	−0.22	0
0.1	9.65	102.98	46.34	9.65	89.7	40.36	92.22
0.2	19.27	95.864	43.04	19.27	97.04	43.67	184.03
0.3	28.9	96.12	43.25	28.9	96.56	43.45	276.01
0.4	38.53	96.32	43.34	38.53	96.36	43.36	368.0
0.5	48.17	96.34	43.35	48.17	96.34	43.35	460.0
0.6	57.8	96.34	43.35	57.8	96.34	43.35	552.0
0.7	67.44	96.34	43.35	67.44	96.34	43.35	644.01
0.8	77.07	96.34	43.35	77.07	96.34	43.35	736.01
0.9	86.67	96.34	43.35	86.67	96.34	43.35	828.01
1	96.34	96.34	43.35	96.34	96.34	43.35	920.01
